# Analysis of the Potential Topical Anti-Inflammatory Activity of *Averrhoa carambola* L. in Mice

**DOI:** 10.1093/ecam/neq026

**Published:** 2011-05-02

**Authors:** Daniela Almeida Cabrini, Henrique Hunger Moresco, Priscila Imazu, Cíntia Delai da Silva, Evelise Fernandes Pietrovski, Daniel Augusto Gasparin Bueno Mendes, Arthur da Silveira Prudente, Moacir Geraldo Pizzolatti, Inês Maria Costa Brighente, Michel Fleith Otuki

**Affiliations:** ^1^Laboratory of Inflammation, Department of Pharmacology, Universidade Federal do Paraná, Curitiba, PR, Brazil; ^2^Department of Chemistry, Universidade Federal de Santa Catarina, Campus Universitário, Trindade, Florianópolis, SC, Brazil; ^3^Department of Pharmaceutical Sciences, Universidade Estadual de Ponta Grossa, Ponta Grossa, Campus Uvaranas, Av. General Carlos Cavalcanti 4748, Bloco M, Sala 94, 84030-900, PR, Brazil

## Abstract

Inflammatory skin disorders, such as psoriasis and atopic dermatitis, are very common in the population; however, the treatments currently available are not well tolerated and are often ineffective. *Averrhoa carambola* L. (Oxalidaceae) is an Asian tree that has been used in traditional folk medicine in the treatment of several skin disorders. The present study evaluates the topical anti-inflammatory effects of the crude ethanolic extract of *A. carambola* leaves, its hexane, ethyl acetate, and butanol fractions and two isolated flavonoids on skin inflammation. Anti-inflammatory activity was measured using a croton oil-induced ear edema model of inflammation in mice. Topically applied ethanolic extract reduced edema in a dose-dependent manner, resulting in a maximum inhibition of 73 ± 3% and an ID_50_ value of 0.05 (range: 0.02–0.13) mg/ear. Myeloperoxidase (MPO) activity was also inhibited by the extract, resulting in a maximum inhibition of 60 ± 6% (0.6 mg/ear). All of the fractions tested caused inhibition of edema formation and of MPO activity. Treatment with the ethyl acetate fraction was the most effective, resulting in inhibition levels of 75 ± 5 and 54 ± 8% for edema formation and MPO activity, respectively. However, treatment of mice with isolated compounds [apigenin-6-*C*-**β**-l-fucopyranoside and apigenin-6-*C*-(2**″**-*O*-**α**-l-rhamnopyranosyl)-**β**-l-fucopyranoside] did not yield successful results. Apigenin-6-*C*-(2**″**-*O*-**α**-l-rhamnopyranosyl)-**β**-l-fucopyranoside caused only a mild reduction in edema formation (28 ± 11%). Taken together, these preliminary results support the popular use of *A. carambola* as an anti-inflammatory agent and open up new possibilities for its use in skin disorders.

## 1. Introduction

The skin is an external organ that covers the entire body surface. It is responsible for the communication between an organism and the environment and is constantly subjected to exogenous stimuli. The main function of the skin is to protect the organism from environmental insults [1, 2]. Fulfilling its role, the skin is able to activate a defense mechanism aimed at pathogen elimination and tissue repair [3]. Initiation of the defense response is characterized by the infiltration of neutrophils and the release of several pro-inflammatory mediators, which starts the inflammatory process. If this inflammatory response is not appropriately regulated, an inflammatory skin disease can be triggered [4]. The most common inflammatory skin disorders include atopic dermatitis and psoriasis. Both of these disorders can have a high impact on the patient's quality of life, and the treatments for these diseases are usually not effective [5–7]. Because currently available therapeutics to treat chronic inflammatory skin diseases are mostly ineffective and produce a plethora of side effects, the search for more effective and safer treatment alternatives is necessary. Natural products derived from plants have long been used in folk medicine, making the compounds derived from these plants good candidates for new therapeutic strategies [8–10].

*Averrhoa carambola* L. (Oxalidaceae) is an Asian tree that was introduced to Brazil. This tree is also known as the star fruit tree and is commonly used to treat headaches, vomiting, coughing and hangovers [11]. Furthermore, it is used as an appetite stimulant, a diuretic, and as an antidiarrheal and febrifugal agent. *A. carambola* has been used in the treatment of eczemas [12]. In addition, the extract obtained through decocting the leaves of *A. carambola* has been used in the treatment of diabetes [13].

Phytochemistry studies have shown that the fruit of *A. carambola* is rich in antioxidants, especially polyphenolic compounds, which act against reactive oxygen species. Investigations characterizing the secondary metabolites of *A. carambola* have identified two *O*-glycosyl flavonoid components: quercetin-3-*O*-*β*-d-glucoside and rutin [14]. Other compounds indentified included the following: *β*-sitosterol, lupeol, anthraquinone glucoside [15], cyanidin-3-*O*-*β*-d-glucoside, cyanidin-3,5-*O*-*β*-d-diglucoside [16], *β*-amirin [17], and C-glycoside flavones, such as apigenin-6-*C*-*β*-l-fucopyranoside and apigenin-6-*C*-(2^*″*^-*O*-*α*-l-rhamnopyranosyl)-*β*-l-fucopyranoside. This latter compound is also known as carambolaflavone [18].

Furthermore, the insoluble fibers of the star fruit slow the absorption of carbohydrates, significantly reducing blood glucose levels. The fiber can also act to prevent cardiovascular disease by reducing serum triglyceride and total cholesterol levels [19–21]. Lastly, selective activity against brain tumor cells was observed with an alcoholic extract from the stems of *A. carambola*, while an extract from the leaves was effective against liver carcinoma cells [17].

In the present study, we investigated the potential anti-inflammatory properties of the leaves from this plant. For these studies, the topical anti-inflammatory activity of the ethanolic extract and two isolated flavonoids from the leaves of *A. carambola* were evaluated on a classic model of skin inflammation—croton oil-induced mouse ear edema.

## 2. Methods

### 2.1. Extraction and Isolation

The leaves of *A. carambola* were collected in March 2003 at Santo Amaro da Imperatriz in Santa Catarina, Brazil, and identified by botanist Daniel de Barcellos Falkenberg. A voucher specimen (FLOR 24.144) was deposited in the Herbarium FLOR, Universidade Federal de Santa Catarina, Santa Catarina, Brazil.

The air-dried leaves of *A. carambola* (281 g) were extracted with 80% ethanol at room temperature for 15 days. The solvent was removed by rotary evaporation (at <55°C). The ethanolic extract (41.3 g) was resuspended in 80% (v/v) ethanol and partitioned with hexane (Hex), ethyl acetate (EtOAc) and *n*-butanol (*n*-BuOH). The EtOAc fraction (6.9 g) was run on a silica gel-based chromatography column and was eluted with mixtures of various ratios of Hex, EtOAc, and ethanol to yield the flavonoids, apigenin-6-*C*-*β*-l-fucopyranoside (150 mg) (compound 1) and apigenin-6-*C*-(-2^*″*^-*O*-*α*-l-rhamnopyranosyl)-*β*-l-fucopyranoside (200 mg) (compound 2). Identification of the flavonoids was carried out using spectral identification methods (1H NMR, 1H–1H COSY, ^13^C NMR, DEPT, HMQC, HMBC) and by comparing these data with those reported in the literature [22, 23].

### 2.2. Animals

Experiments were performed on Swiss male mice (25–35 g) housed at 22 ± 2°C under a 12-h light/12-h dark cycle, with free access to food and water. Experiments were performed during the light phase of the cycle. The animals were allowed to adapt to the laboratory for at least 1 h before testing and were used only once. The experiments reported in this study were carried out in accordance with guidelines specified by the Ethics Committee on Animal Experimentation of the Federal University of Paraná and are in accordance with international guidelines.

### 2.3. Ear Edema Measurements

Edema was quantified by the increase in ear thickness of mice upon inflammatory challenge. Ear thickness was measured before and after induction of the inflammatory response using a digital micrometer (Great, MT-045B). The micrometer was applied near the tip of the ear just distal to the cartilaginous ridges, and the thickness was recorded in micrometers. To minimize technique variations, a single investigator performed the measurements throughout each experiment [24]. The phlogistic agents, the ethanolic extract and its fractions and compounds were dissolved in 20 *μ*L of acetone and applied to the right ear of each mouse.

### 2.4. Croton-Oil-Induced Ear Edema

Edema was induced in the right ear by topical application of croton oil at a concentration of 0.4 mg/ear. Hex, EtOAc and BuOH fractions of the ethanolic extract, compounds 1 and 2 from *A. carambola*, and dexamethasone (DE) (a positive control) were applied topically immediately after croton oil treatment. Ear thickness was measured prior to and 6 h after the induction of inflammation. Ear samples (circles of tissue 6 mm in diameter) were collected 24 h after the application of croton oil and were measured for myeloperoxidase (MPO) activity.

### 2.5. Tissue MPO Activity Assay

The activity of tissue MPO was assessed 24 h after croton oil application to the mouse ear according to the technique reported by Suzuki et al. [25] and modified by De Young et al. [26]. A biopsy (6 mm ear tissue punch) was placed in 0.75 mL of 80 mM phosphate-buffered saline (PBS), pH 5.4 containing 0.5% hexadecyltrimethylammonium bromide and homogenized (45 s at 0°C) in a motor-driven homogenizer. The homogenate was decanted into a microfuge tube, and the vessel was washed with a second 0.75 mL aliquot of hexadecyltrimethylammonium bromide in buffer. The wash was added to the tube, and the 1.5 mL sample was centrifuged at 12 000 g at 4°C for 15 min. Samples of the resulting supernatant were added to 96-well microlitre plates in triplicate at a volume of 30 *μ*L. For the MPO assay, 200 *μ*L of a mixture containing 100 *μ*L of 80 mM PBS pH 5.4, 85 *μ*L of 0.22 M PBS pH 5.4 and 15 *μ*L of 0.017% hydrogen peroxide were added to the wells. The reaction was started by addition of 20 *μ*L of 18.4 mM tetramethylbenzidine HCl in dimethylformamide. The plates were incubated at 37°C for 3 min and then placed on ice. The reaction was stopped by the addition of 30 *μ*L of 1.46 M sodium acetate, pH 3.0. Enzyme activity was determined colorimetrically using a plate reader (EL808-BioTech Instruments, Inc., Winooski, VT, USA) set to measure absorbance at 630 nm and is expressed as mOD mg per tissue.

### 2.6. Drugs

The following substances were used: croton oil, DE, hexadecyltrimethylammonium bromide, tetramethylbenzidine, hydrogen peroxide (all from Sigma-Aldrich, St. Louis, MO, USA), sodium acetate, dimethylformamide, acetone and absolute ethanol (all from Merck, Darmstadt, Germany).

### 2.7. Statistical Analysis

The results are presented as mean ± SEM with exception of the ID_50_ values (dose required to reduce the responses of the treated groups by 50% relative to the control group), which are reported as geometric means plus their respective 95% confidence limits. The statistical significance between the groups was assessed by one-way analysis of variance (ANOVA) followed by a post hoc Newman-Keuls test. The accepted level of significance for the test was *P* < .05. All tests were carried out using GraphPad Software (San Diego, CA, USA).

## 3. Results

### 3.1. *Averrhoa carambola* on Croton Oil-Induced Cutaneous Inflammation

**Figure 1:**
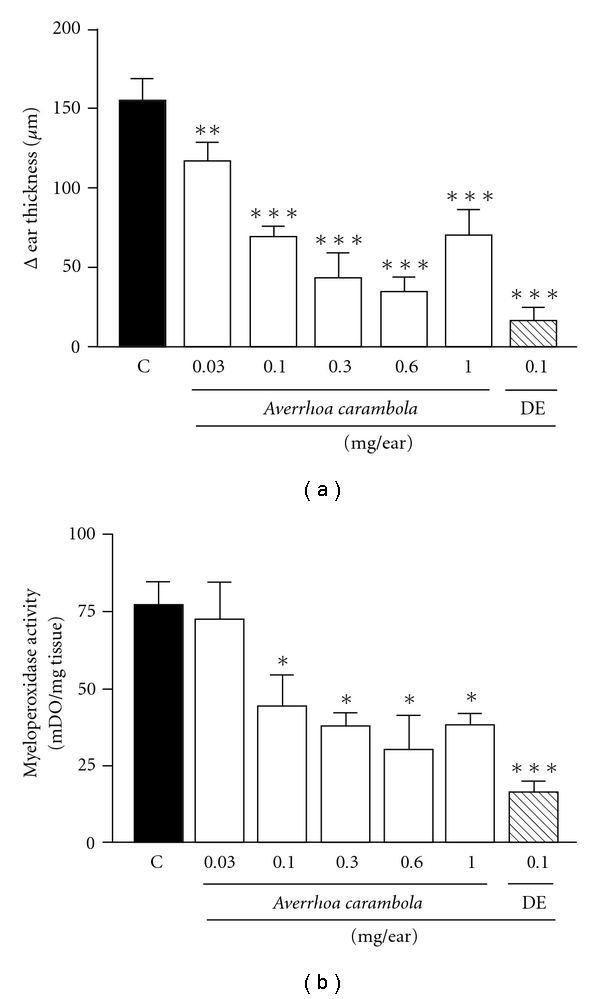
Effect of ethanolic extract from *A. carambola* and DE topically administered on croton oil-induced ear edema (a) and MPO activity (b). Ear edema and enzymatic activity was measured at 6 h and 24 h after croton oil treatment, respectively. The extract (0.03–1.0 mg/ear) and DE (0.1 mg/ear) were applied after croton oil application. Each bar represents the mean ± SEM for four to five animals. The graphic symbols denote the significance levels when compared with control groups. Significantly different from controls, *P < .05, **P < .01, ***P < .001.

Topical application of croton oil promoted an increase in the thickness of the ear and in the tissue MPO activity. Upon application of the ethanolic extract of *Averrhoa* or the various fractions of the extract, croton oil-induced ear edema and cellular migration in mice were both reduced effectively. As shown in Figure 1(a), topically applied ethanolic extract from *A. carambola* (0.03–1.0 mg/ear) resulted in a dose-dependent inhibition of croton oil-induced ear edema, with an ID_50_ value of 0.05 (0.02–0.13) mg/ear and a maximum inhibition of 78 ± 5% (at 0.6 mg/ear). MPO is a marker of polymorphonuclear leukocytes. MPO activity is directly related to the amount of leukocyte infiltration, which is indicative of an inflammatory reaction. In order to verify the effects of the extract on croton oil-induced cell infiltration, MPO activity was assessed. Ethanolic extract treatment (0.03–1.0 mg/ear) promoted a dose-dependent reduction in enzyme activity (Figure 1(b)). The maximum inhibition was 61 ± 16% (at 0.6 mg/ear), and the ID_50_ value was 0.22 (0.08–0.60) mg/ear. In these tests, treatment with the reference drug, DE (at 0.1 mg/ear), resulted in an inhibition of edema and MPO activity by 89 ± 5 and 79 ± 4%, respectively.

### 3.2. Activity of Fractions from the Ethanolic Extract of *A. carambola*

**Figure 2:**
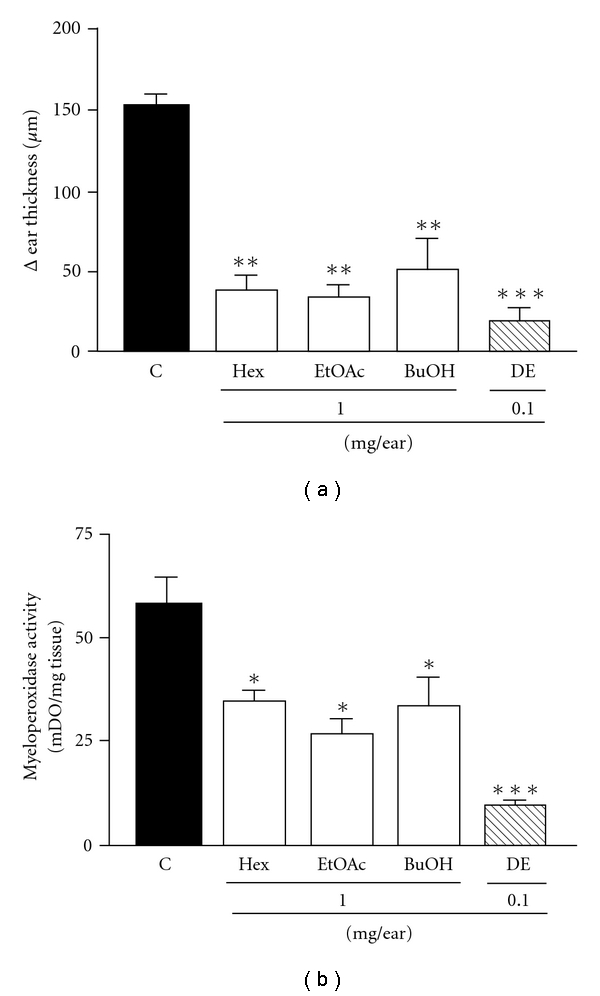
Effect of the fractions of ethanolic extract from *A. carambola* and DE administered topically on croton oil-induced ear edema (a) and MPO activity (b). Ear edema and enzymatic activity was measured at 6 h and 24 h after croton oil treatment, respectively. Animals were challenged with croton oil and so treated with the fractions Hex, EtOAc and BuOH (1.0 mg/ear) and DE (0.1 mg/ear). Each bar represents the mean ± SEM for four to five animals. The graphic symbols denote the significance levels when compared with control groups. Significantly different from controls, *P < .05, **P < .01, ***P < .001.

In view of the results with the extract, we further investigated whether its fractions could change these inflammatory parameters. Hex, EtOAc and BuOH fractions from the ethanolic extract of *A. carambola* (1.0 mg/ear) also reduced ear edema formation. Treatment resulted in maximum inhibition values of 73 ± 7, 75 ± 5 and 63 ± 14% for the Hex, EtOAc and BuOH fractions, respectively (Figure 2(a)).  Figure 2(b) shows the MPO inhibition caused by the *A. carambola* fractions (at 1.0 mg/ear). All of the fractions were able to reduce the croton oil-induced increase in enzyme activity, with inhibition values of 40 ± 4, 54 ± 8 and 42 ± 11% using the Hex, EtOAc and BuOH fractions, respectively. Once again, DE treatment caused an inhibition of 86 ± 7 and 83 ± 1% of edema and cell migration, respectively.

### 3.3. Evaluation of Flavonoids Isolated from the *A. carambola* Extract

**Figure 3:**
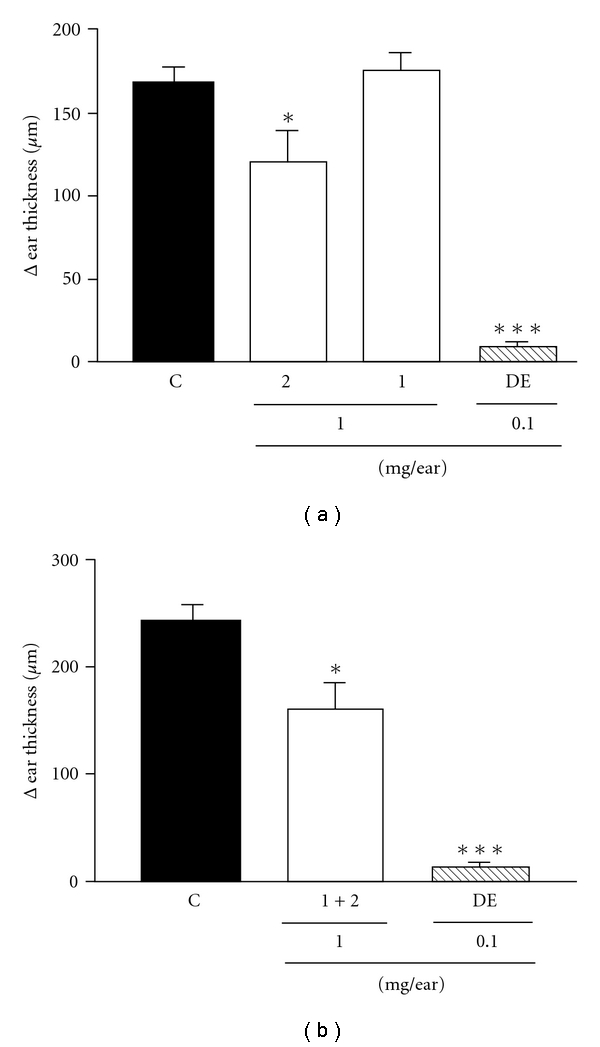
Effect of isolated compounds from *A. carambola* and DE topically administered on croton oil-induced ear edema (a) and the effect of interaction between the compounds (b). Ear edema was measured at 6 h after croton oil treatment. Animals were challenged with croton oil and so treated with the compounds 1 (apigenin-6-*C*-*β*-l-fucopyranoside) and 2 (apigenin-6-*C*-(-2^*″*^-*O*-*α*-l-rhamnopyranosyl)-*β*-l-fucopyranoside) (1.0 mg/ear) or DE (0.1 mg/ear). Each bar represents the mean ± SEM for four to five animals. The graphic symbols denote the significance levels when compared with control groups. Significantly different from controls, *P < .05, ***P < .001.

Since the ethanolic extract from *A. carambola* and its fractions were effective in reducing the inflammatory parameters, it became interesting to analyze the effects of topical application of the isolated flavonoids, compounds 1 and 2. As shown in Figure 3(a), topically applied compound 1 (1.0 mg/ear) did not alter the croton oil-induced ear edema, while flavonoid 2 caused a mild inhibition (28 ± 11%) at a dose of 1.0 mg/ear. DE inhibited edema by 95 ± 2%. Among natural compounds, interaction effects are often observed. In order to determine whether there is a possible interaction between the isolated compounds, another experiment was performed in which the two compounds were co-administered. The inhibition of edema formation observed upon co-administration was 34 ± 11%, which was identical to the inhibition caused by compound 2 alone. Once again, DE resulted in an inhibition of 95 ± 2% (Figure 3(b)).

## 4. Discussion

Acute inflammation is characterized by classical symptoms, such as heat, redness, swelling and pain. Edema (swelling) is therefore a good measure of inflammation and is useful for the quantification of skin inflammation induced by phlogistic agents such as croton oil. Croton oil-induced ear edema is a widely used method for studying the inflammatory process in skin, and for identifying anti-inflammatory agents that could be useful in the treatment of skin disorders [26, 27].

The present study provides evidence that *A. carambola* leaves have a relevant topical anti-inflammatory effect in a model of cutaneous inflammation in mice. Here, we showed that the plant reduced edema and inhibited the cellular migration of polymorphonuclear leukocytes, an important step in the inflammatory process. Croton oil is a phlogistic agent extracted from *Croton tiglium* L., Euphorbiaceae, and it has an irritant and vesiculant effect on the skin. Croton oil contains phorbol esters, being the 12-*O*-tetradecanoylphorbol-13-acetate (TPA) the predominant phorbol ester. Topical application of croton oil or TPA promotes an acute inflammatory reaction characterized by vasodilatation, polymorphonuclear leukocyte infiltration to the tissue and edema formation. These changes are triggered by protein kinase C (PKC) activation, which promotes an increase in the activity of phospholipase A_2_ (PLA_2_). Activation of PLA_2_ results in increased levels of arachidonic acid and its metabolites, such as prostaglandins and leukotrienes [24, 27–29]. Moreover, PKC also promotes the secretion and activation of several immune mediators such as cytokines and chemokines which increase and maintain the skin inflammatory response [30].

The ethanolic extract and its fractions promoted a significant and dose-dependent inhibition of croton oil-induced skin inflammation. Topically applied croton oil resulted in activation of pro-inflammatory mediators that promoted the manifestation of several inflammatory parameters similar to some skin disorders [31]. Using this model, compounds that inhibit this process, such as the ones in *A. carambola*, can be target in the search for new therapeutic strategies.

Unlike the ethanolic extract, which showed efficient anti-edematogenic activity, the isolated flavonoids did not demonstrate pronounced activities toward reducing ear edema. Compound 2 caused mild inhibition of edema, while compound 1 had no effect. In the field of herbal medicine, interaction of the activities of compounds found in plant extracts may result in the potentiation of the activity of each compound by the others. This could explain why numerous attempts to isolate individual active compounds from medicinal plants have been unsuccessful [32]. In order to determine whether there is an interaction between our two compounds of interest, we evaluated the effects of co-administering them on inflammation. The effect of co-administering the compounds was not different from those observed upon administration of compound 2 alone. These results suggest that these compounds are not responsible for the ethanolic extract anti-inflammatory response.

Cellular infiltration represents an important feature in skin inflammation, and neutrophils are the predominant type of cells that infiltrate the area. These cells play a crucial role in cutaneous inflammation. Leukocyte accumulation in the skin is important for the progression of the inflammatory reaction as well as for the increase in expression of some inflammatory enzymes such as cyclooxygenase-2 [33, 34]. MPO is an enzyme known to be a marker of neutrophil infiltration. Thus, inhibition of MPO activity can be used to indicate the presence of an anti-inflammatory reaction [4, 35]. Topical treatments with the ethanolic extract or its fractions were able to inhibit MPO activity, indicating that these compounds may influence cell migration during the inflammatory process. However, it is too early to propose a detailed mechanism through which the extract/fractions exert their anti-inflammatory activity. *Averrhoa carambola* compounds could be influencing one or more steps of the croton oil-induced inflammatory cascade, such as protein kinase C and phospholipase A_2_ activation, cyclooxygenase-2 induction, and cytokine production and release [26, 27].

Nowadays, the treatment of inflammatory skin diseases is very difficult because these diseases are chronic and need extended treatment, which can result in the development of drug resistance by the patient. The most common treatments used are based on corticosteroids [36], and recently, treatments such as monoclonal antibodies against immunoglobulin E as efalizumab have been used [37, 38]. However, these treatments are often ineffective and/or accompanied by a series of undesirable side effects [39]. Therefore, research has currently been focused on finding effective methods to interfere with the inflammatory cascade. An effective measure would be the inhibition of nuclear factor kappa B (NF-*κ*B). This transcription factor regulates the transcription of important pro-inflammatory genes, such as genes for cytokines, chemokines, adhesion molecules, COX-2, nitric oxide synthase and others. Thus, the inhibition of NF-*κ*B prevents the production of these mediators, resulting in a decrease in the inflammatory process [40]. A large group of natural substances has demonstrated anti-inflammatory activity. Among these substances are the following: flavonoids, tannins, steroids and terpenes, which are able to interfere with several components of the inflammatory cascade [41]. As previously shown, *A. carambola* is rich in these kinds of compounds, particularly with flavones. Although the flavonoids evaluated in this study were ineffective, it would be interesting to determine the potential activity of the other compounds from the plant in order to elucidate the mechanism by which the plant influences the inflammatory process.

**Figure 4:**
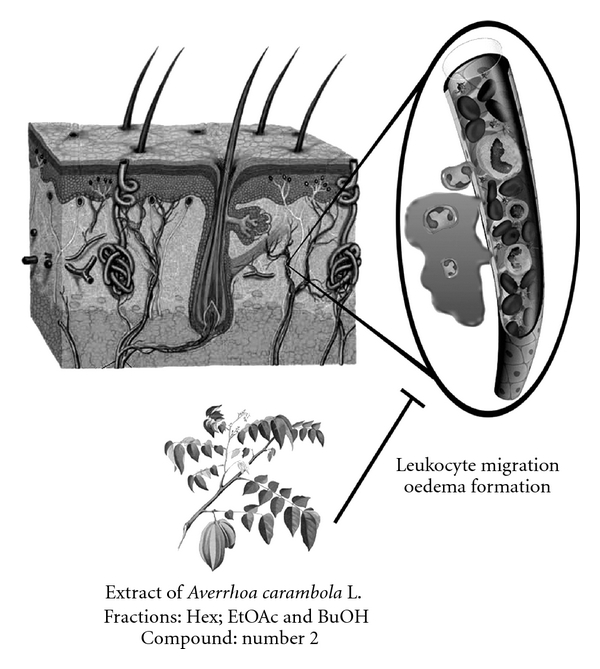
Proposed effect of *A. carambola* against croton oil-induced skin responses. Schema represents a cross section of skin showing its layers—epidermis, dermis and hypodermis, as well as skin appendages (hair follicle, sudoriferous and sebaceous glands). Highlights a capillary which after croton oil stimulus promotes plasma leakage and cell migration, and *A. carambola* extract, fractions and compound 2 inhibit both events (cell migration and edema).

In summary, we have shown that the ethanolic extract from *A. carambola* and its BuOH, EtOAc and Hex fractions are effective in reducing croton oil-induced ear edema and cellular migration in mice (Figure 4). Although additional studies are necessary to address the mechanism of action of *A. carambola*, our results support the popular use of this plant for skin inflammatory disorders.

## Funding

Grants from the Conselho Nacional de Desenvolvimento Científico e Tecnológico (CNPq, Brazil) and from the Programa de Apoio a Planos de Reestruturação e Expansão das Universidades Federais (REUNI)/Coordenação de Aperfeiçoamento de Pessoal de Nível Superior (CAPES, Brazil).
